# Reciprocal interactions between lncRNAs and MYC in colorectal cancer: partners in crime

**DOI:** 10.1038/s41419-024-06918-w

**Published:** 2024-07-29

**Authors:** Zhen Lei, Zhipu Zhu, Zhihui Yao, Xiangyu Dai, Yi Dong, Bing Chen, Songyu Wang, Siyue Wang, Lutterodt Bentum-Ennin, Lei Jin, Hao Gu, Wanglai Hu

**Affiliations:** 1 Translational Research Institute, People’s Hospital of Zhengzhou University, Academy of Medical Science, Henan International Joint Laboratory of Non-coding RNA and Metabolism in Cancer, Tianjian Laboratory of Advanced Biomedical Sciences, State Key Laboratory of Esophageal Cancer Prevention and Treatment, Zhengzhou University, Zhengzhou, 450003 China; 2 Department of Immunology, School of Basic Medical Sciences, Anhui Medical University, Hefei, 230027 China

**Keywords:** Oncogenes, Long non-coding RNAs

## Abstract

Proto-oncogenic *MYC* is frequently dysregulated in colorectal cancer (CRC). In the past decades, long noncoding RNAs (lncRNAs) have emerged as important regulators in cancers, acting as scaffolds, molecular decoys, post-transcriptional regulators, and others. Interestingly, lncRNAs are able to control MYC expression both at transcriptional and post-transcriptional levels. It is suggested that the reciprocal interaction of MYC and lncRNAs often occurs in CRC. MYC can affect the cell fate by promoting or inhibiting the transcription of some lncRNAs. At the same time, some lncRNAs can also affect MYC expression or transcriptional activity, and in turn decide the cell fate. In this review we summarized the current knowledge about the MYC and lncRNA axis, focusing on its mutual regulation, roles in CRC, and proposed potential therapeutic prospects for CRC treatment.

## Facts

- MYC is responsible for regulating a plethora of genes directing a range of molecular functions.
- A deregulated expression and/or activity of MYC is a common observation in CRC.
- LncRNAs are emerging as important factors in cancers by regulating gene expression on multiple levels.
- Both MYC and lncRNAs have been elusive targets clinically. Recently, however, the discovery of novel inhibitors, as well as, new forms of administration have brought new hope to the search for therapies targeting MYC and lncRNAs.

## Open questions

- How do we limit the pro-oncogenic function of lncRNAs and MYC more effectively? Should we target lncRNAs or disrupt the interaction between lncRNAs and MYC?
- Will the interaction between MYC and lncRNAs differ in other tumors besides colorectal cancer?
- Is there a similar relationship between lncRNAs and MYC in immune cells? Can targeting MYC or lncRNAs advance the course of cancer immunotherapy?

## Introduction

Advanced colorectal cancer (CRC) is a pervasive cancer with a high mortality rate. Worldwide in 2022, 1.9 million people were diagnosed with CRC with 904,000 lives being lost to this disease [1]. Unfortunately, the tide of early-onset CRC is rising, and these younger individuals are presenting with predominantly distal tumors, a site of occurrence known to present with a worsened prognosis [2, 3]. Obesity, heredity, and diet contribute to the CRC development [4] with smoking also considered as a major risk factor for CRC [5]. As with most sporadic cancers, individual cases of CRC are highly heterogenous, with several studies identifying the dysregulation of multiple genes arising from a range of genetic and epigenetic causes that affect both coding and noncoding genes [6–10].

One of the most common changes occurring in CRC and over 70% of other cancers involves the abnormal expression and/or activity of MYC [11], attracting a great deal of research attention. MYC is a transcription factor belonging to the MYC family [12] and is responsible for regulating the transcription of a plethora of genes directing a range of molecular functions. Notably, MYC is not only essential for maintaining normal cellular homeostasis where its expression is strictly regulated but its dysregulation appears to be equally important for tumor development and progression. Indeed, evidence continues to build up linking MYC with the control of hallmark metabolic pathways in cancer cells including glycolysis, glutaminolysis, oxidative phosphorylation, and lipid metabolism [13–16]. Such metabolic reprogramming is achieved through control of many of the key rate-limiting enzymes in these pathways. In turn, this emphasizes the importance of dissecting the “life cycle” of MYC in cancer, understanding its key downstream targets, and how MYC itself is dysregulated.

Long noncoding RNAs (lncRNAs) are noncoding RNAs >200nt, which lack coding potential but regulate gene expression at multiple levels, acting through binding to DNA, RNA and proteins to exert effects through epigenetic, transcriptional, translational, and post-translational levels. Many lncRNAs play key roles in processes crucial to cancer cell survival including metabolism, cell cycle, apoptosis, and differentiation [17–20]; however, the fact remains that currently, the function and mechanism of only a relatively few lncRNAs have been illustrated in detail [21–23]. Aside from the functions listed above, lncRNAs have also been reported to serve as scaffolds for RNA-binding proteins, therefore facilitating liquid-liquid phase separation and maintaining genomic stability [24–26].

In this review, we elaborate on the regulatory networks between MYC and lncRNAs, using CRC as an exemplary cancer model. The first section describes a series of lncRNAs known to be regulated by MYC, with emphasis on the mechanistic details involving both transcriptional and post-transcriptional regulation. We then discuss how lncRNAs are involved in the reciprocal regulation of MYC (both positively and negatively). As noted above, MYC expression must be tightly controlled, and interestingly, MYC-lncRNA connections often incorporate regulatory feedback loops as illustrated by several examples described below. We then conclude by citing recent applications and prospects for exploiting the MYC-lncRNA axis in the diagnosis and treatment of CRC. In short, this review aims to provide readers with an objective overview together with a comprehensive understanding of the reciprocal interactions between MYC and lncRNAs in CRC.

## LncRNAs driven by MYC

One of the reported functions of lncRNAs involves their role as competitive endogenous RNAs (ceRNAs) which serve to recruit regulatory microRNAs (miRNAs), thus sponging and decoying miRNAs away from their intended mRNA targets [27]. Indeed, several reports have now shown that MYC transcript levels are controlled via this mechanism [28]. For example, lncRNA-MIF (MYC inhibitory factor), acts as a tumor suppressor to dampen MYC expression in CRC and other cancers, in turn inhibiting aerobic glycolysis and tumorigenesis enacted through MYC. This regulation is established by lncRNA-MIF sequestering miR-586 to alleviate its binding to FBXW7 mRNA transcripts, the latter being an E3-ligase targeting MYC, with the resultant increase in FBXW7 expression responsible for MYC degradation. Given that lncRNA-MIF is itself transactivated by MYC, this creates a regulatory feedback loop between lncRNA-MIF and MYC [29–31].

LncRNAs are multifunctional and play different roles as scaffolding molecules, binding to chromatin and/or proteins to alter effector recruitment. Here, SENEBLOC, a MYC-responsive lncRNA, provides an example of different modalities involving ceRNA and scaffolding mechanisms. SENEBLOC prevents CRC cells from triggering cell senescence by preventing the induction of the senescence initiator, *CDKN1A*. Firstly, SENEBLOC functions as a ceRNA with miR-3175, stabilizing HDAC5 expression which results in epigenetic silencing at the *CDKN1A* gene promoter. Secondly, SENEBLOC binding facilitates p53 associating with MDM2, promoting p53 turnover and decreasing *CDKN1A* transactivation [32]. Thus, SENEBLOC contributes to a dual mechanism that converges to regulate *CDKN1A* expression. Another intriguing example of scaffolding involves the lncRNA glycoLINC, also named USP2-AS1. GlycoLINC was shown to assemble a metabolon complex between five glycolytic enzymes, namely PGK1, PGAM1, ENO1, PKM, and LDHA. In CRC cells with limited access to serine, glucose-derived carbon is shunted towards the serine synthesis pathway while metabolon assembly serves to increase glycolytic efficiency to help preserve vital ATP levels and enable cancer cells to survive [33].

MYC-regulated lncRNAs also serve to promote the expression of a substantial list of oncogenes by regulating their mRNA stability, both in direct and indirect manners. The MYC-activated lncRNA LAST (lncRNA-assisted stabilization of transcripts) positively regulates *CCND1* (cyclin D1) mRNA stability to promote tumorigenesis. LAST cooperates with the single-stranded DNA/RNA-binding factor CNBP to prevent the degradation of *CCND1* mRNA [34]. Another transcriptional target of MYC is MYU, a lncRNA commonly upregulated in CRC. MYU associates with the RNA-binding protein HNRNPK and this complex occupies the *CDK6* gene promoter to stabilize CDK6 transcription and expression to drive CRC cells through the cell cycle [35].

**Fig. 1:**
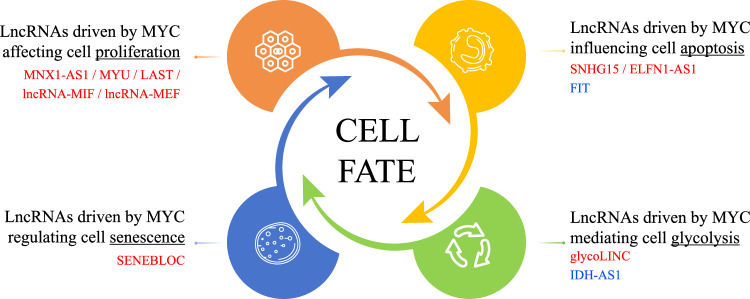
LncRNAs driven by MYC. MYC can decide cell fate by regulating lncRNAs. For instance, MNX1-AS1, MYU, LAST, lncRNA-MIF, lncRNA-MEF can be regulated by MYC, and therefore affect cell proliferation. MYC can influence apoptosis by regulating SNHG15, ELFNI-AS1 and FIT. Also, glycoLINC and IDH-AS1, two MYC-responsive lncRNAs can bind to metabolic enzymes and effect glycolysis. SENEBLOC binding facilitates the association of p53 with MDM2, thus regulating CDKN1A expression and eventually influencing cell senescence. LncRNAs in red font are MYC transcription-activated lncRNAs, while lncRNAs in blue font are MYC transcription-repressed lncRNAs.

Another functional twist involves the effects on protein activity following lncRNA binding. The MYC-regulated lncRNA SNHG15 interacts with AIFM1 (Apoptosis Induced Factor Mitochondria Associated 1) to promote the oncogenic phenotype in CRC cells, suggesting SNHG15 acts partially by regulating the activity of AIFM1. Interestingly, ROS levels, which are directly regulated by AIFM1, also show significant reductions in SNHG15-depleted cells. Knockdown of SNHG15 significantly promotes the sensitivity of CRC cell lines treated with 5-FU [36]. In a similar fashion, the proliferation of CRC cells is facilitated by the MYC-regulated lncRNA MNX1-AS1 that binds to activate the Y-box-binding protein 1 (YBX1) [37]. Furthermore, one of the most interesting examples of the lncRNA-MYC regulatory pathway involves the lncRNA generated from the antisense region of isocitrate dehydrogenase 1 (IDH1-AS1). IDH1-AS1 binding promotes the homodimerization and activation of the IDH1 enzyme, promoting glycolysis in concert with influencing HIF1α activation, key features of the Warburg effect. Nonetheless, IDH1-AS1 expression is subject to MYC repression to control this circuit [38]. Lastly, the lncRNA-MEF (MYC Enhancing Factor) was found to protect HNRNPK from ubiquitination and proteasomal destruction, inhibiting its association with the E3-ligase TRIM25. In turn, this facilitates MYC expression resulting from HNRNPK promoting the translation of MYC [39]. A current list of relevant MYC-regulated lncRNAs [40, 41] is illustrated in Fig. 1 and Table 1, respectively. In summary, lncRNAs driven by MYC in CRC can promote or inhibit cell growth mainly by acting as scaffolds, sponging miRNAs, directly affecting downstream mRNA or protein, and consequently deciding cell fate.

**Table 1:** LncRNAs driven by MYC.

lncRNA driven by MYC	Activated/Repressed	Locus	Cellular process	Ref.
LncRNA-MIF	Activated	Cytoplasm	Cell cycle	[29]
LAST	Activated	Cytoplasm	Cell cycle	[34]
MYU	Activated	Cytoplasm	Cell cycle	[35]
MNX1-AS1	Activated	Cytoplasm & Nucleus	Cell cycle	[37]
LncRNA-MEF	Activated	Cytoplasm & Nucleus	Cell cycle	[39]
SNHG15	Activated	Cytoplasm	Apoptosis	[36]
FIT	Repressed	Nucleus	Apoptosis	[40]
ELFN1-AS1	Activated	Cytoplasm	Apoptosis	[41]
SENEBLOC	Activated	Cytoplasm & Nucleus	Senescence	[32]
GlycoLINC	Activated	Cytoplasm	Glycolysis	[33]
IDH-AS1	Repressed	Cytoplasm	Glycolysis	[38]

## LncRNAs regulating MYC transcription

**Fig. 2:**
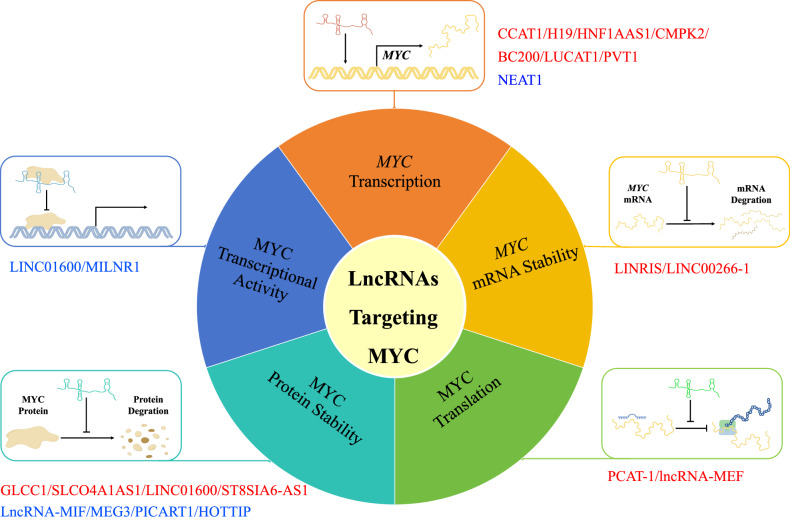
LncRNAs mediated regulation of MYC. LncRNAs play a regulatory role in multiple physiological processes of MYC, including transcriptional, post-transcriptional, and protein function stage. CCAT1, H19, HNF1A-AS1, CMPK2, BC200, LUCAT1, PVT1, and NEAT1 can bind to transcription-associated proteins and influence MYC transcription. At the post-transcriptional level, LINC00266-1 and LINRIS can affect *MYC* mRNA stability; PCAT-1 and lncRNA-MEF can affect MYC at translational level; besides, MYC protein stability can be affected by GLCC1, SLCO4A1-AS1, LINC01600, ST8SIA6-AS1, lncRNA-MIF, MEG3, PICART1 and HOTTIP. In addition to affecting expression level of MYC, lncRNAs can also affect the transcriptional activity of MYC. MILNR1 can bind with MYC to inhibit the transcription of *NUP88*, a MYC transcriptional-responsive gene. In addition, LINC01600 assists MYC binding to the enhancer region of *FHIT* to suppress *FHIT* activation. The lncRNAs in red font are those that positively regulate MYC, while the lncRNAs in blue font are those that negatively regulate MYC.

LncRNAs can act to alter the recruitment of transcription factors to the *MYC* promoter. The lncRNA LUCAT1 putatively forms G-quadruplex structures that can directly bind to nucleolin (NCL) and interfere with NCL-mediated inhibition of MYC, thereby leading to MYC upregulation [42]. Other lncRNAs regulate MYC transcription through upstream effects. The lncRNA H19 regulates Wnt/β-catenin signaling by targeting PGRN, consequently reducing MYC expression which is downstream of the Wnt/β-catenin pathway [43]. The transcriptional status of the *MYC* gene has been shown to be modulated through lncRNAs acting through different mechanisms. Some lncRNAs can interact with transcriptional factors, thereby influencing the transcription of MYC. Located 515 kb upstream of the *MYC* locus, the lncRNA CCAT1 (colorectal cancer-associated transcript 1), is a nuclear-retained lncRNA reported to establish a super-enhancer locus that augments MYC transcription. CCAT1 functions in concert with the transcription factor CTCF to promote long-range chromatin loops between the *MYC* promoter and the super-enhancer elements [44, 45]. These and other additional lncRNAs involved in regulating MYC transcription [46–50] are summarized in Fig. 2 and Table 2. In summary, nuclear lncRNAs can influence MYC transcription by binding to its promoter region or interacting with other transcriptional regulators which may lead to a change in the transcription of the *MYC* gene.

**Table 2:** LncRNAs targeting MYC at transcription level.

LncRNAs targeting MYC	Locus	Up/Downregulate MYC	Ref.
LUCAT1	Nucleus	Enhances MYC expression	[42]
H19	Not mentioned	Enhances MYC expression	[43]
CCAT1	Nucleus	Enhances MYC expression	[44, 45]
CMPK2	Nucleus	Enhances MYC expression	[46]
NEAT1	Nucleus	Represses MYC expression	[47]
BC200	Nucleus	Enhances MYC expression	[48]
HNF1A-AS1	Not mentioned	Enhances MYC expression	[49]
PVT1	Nucleus	Enhances MYC expression	[50]

## Post-transcriptional control of MYC expression by lncRNAs

In addition to the significant number of lncRNAs regulating MYC transcription, others exert control over MYC expression through post-transcriptional mechanisms. A study from the 1980s showed that the inhibition of protein synthesis controls the degradation of *MYC* mRNA, thus regulating the expression levels of MYC [51]. More recent research has shown that also lncRNAs regulate the expression levels of MYC, and thus tumor progression, through various mechanisms, including mRNA stability, mRNA translation, and protein degradation [29, 52, 53], summarized in Fig. 2 and Table 3.

**Table 3:** LncRNAs targeting MYC at post transcription level.

LncRNAs targeting MYC	Locus	Regulation of MYC	Ref.
LINRIS	Cytoplasm	Stabilizes *MYC* mRNA	[55]
LINC00266-1	Nucleus	Stabilizes *MYC* mRNA	[57]
PCAT-1	Cytoplasm	Enhances MYC translation	[58–60]
LncRNA-MEF	Cytoplasm & Nucleus	Enhances MYC translation	[39]
LncRNA-MIF	Cytoplasm	Represses MYC protein stability	[29]
MEG3	Not mentioned	Represses MYC protein stability	[63]
GLCC1	Cytoplasm	Enhances MYC protein stability	[64]
LINC01600	Cytoplasm	Enhances MYC protein stability	[65]
PICART1	Cytoplasm	Represses MYC protein stability	[67, 68]
HOTTIP	Nucleus	Represses MYC protein stability	[69]
SLCO4A1-AS1	Cytoplasm	Enhances MYC protein stability	[70]
ST8SIA6-AS1	Nucleus	Enhances MYC protein stability	[71]

The abundance of any mRNA is strictly dictated by its half-life and previous studies have shown that lncRNAs can regulate the stability of *MYC* mRNA [54]. The 249-nucleotide coding region instability determinant (CRD) within *MYC* mRNA has been identified as a destabilizing element. However, when this region is bound by CRD-binding proteins (CRDBPs), such as IGF2BP1 and IGF2BP2, it significantly enhances the stability of *MYC* mRNA. The interaction between CRDBPs and *MYC* mRNA, facilitated by lncRNA, indirectly contributes to the stabilization of *MYC* mRNA. The lncRNA LINRIS was shown to block the ubiquitination and degradation of IGF2BP2, an RNA binding and m6A reader protein that binds to m6A-modified *MYC* mRNA to maintain its stability [55]. In addition, lncRNAs can regulate MYC through their encoded polypeptides. Notably, by definition lncRNAs are noncoding but it is emerging that many contain open reading frames that are translated into functional polypeptides [56]. For instance, LINC00266-1 encodes an RNA binding regulatory peptide (RBRP) which binds to IGF2BP1, enhancing IGF2BP1 recognition of m6A-modified *MYC* mRNA, thereby promoting *MYC* mRNA stability [57]. This example shows that lncRNAs can regulate *MYC* mRNA stability not only by employing proteins but also by encoding functional small peptides.

Certain lncRNAs have been shown to regulate *MYC* translation in cancer types other than CRC, although similar phenotypes suggest these mechanisms also operate in CRC cells. For instance, inhibiting PCAT-1 expression in CRC suppresses *MYC* mRNA expression [58], which may be explained by findings in prostate cancer where PCAT-1 regulates the binding between miR-34A and the 3 ‘untranslated region (UTR) of *MYC* mRNA, thus reducing its translation [59, 60]. This demonstrates how lncRNAs may utilize both proteins and other noncoding RNAs in their functional processes to modulate MYC expression at the post-transcriptional level.

The half-life of MYC is approximately 30 min in growing cells [61], lncRNAs may regulate MYC protein levels either through ubiquitination-mediated mechanisms or by directly influencing protein stability. The ubiquitin-mediated proteasomal pathway is primarily responsible for the degradation of MYC [62], and in this context, lncRNAs also contribute to the regulation of MYC levels by influencing protein ubiquitination. In CRC cells, the lncRNA MEG3 inhibits MYC expression by increasing the expression of FBXW7, an E3 ubiquitin ligase that mediates MYC polyubiquitination and degradation [63]. Furthermore, the lncRNA GLCC1 promotes glycolytic flux in CRC cells by stabilizing MYC, forming a stable complex between HSP90AA1 and MYC, preventing MYC polyubiquitination and thus its protein levels [64]. Lastly, a recent study showed that the lncRNA LINC01600 physically interacts with MYC protein in concert with the RBP EIF2S2 to decrease MYC ubiquitination levels and enhance its stability [65].

MYC stability can also be regulated through lncRNAs-mediated post-translational modifications [66]. LncRNAs can also dictate the activation state of GSK3B to modulate phosphorylation levels in MYC at the key regulatory serine 62 (S62) and threonine 58 (T58) residues, thereby affecting stability. Here PICART1, a TP53-induced lncRNA that regulates the AKT2/GSK3B pathway, increases and decreases T58 and S62 residue phosphorylation, respectively, inducing MYC polyubiquitination and directing the protein for destruction [67, 68]. Another lncRNA, HOTTIP, can also regulate the expression levels of GSK3B and MYC, with this mechanism suggested to regulate MYC protein stability [69]. Another lncRNA is known for altering MYC S62 residue phosphorylation levels through the HSP90AA1/CDK2 axis. The lncRNA SLCO4A1-AS1 functions as a molecular scaffold that strengthens the interactions between HSP90AA1 and CDK2, promoting CDK2 protein stability which in turn, stabilizes MYC by promoting S62 phosphorylation [70]. Besides, a novel KRAS-inhibitor-resistant related lncRNA ST8SIA6-AS1, has been found to play a pivotal role in resistance to KRASG12C inhibitors in KRASG12C mutant cell lines through phosphorylation of MYC. Briefly, overexpression of ST8SIA6-AS1 mainly acts as a scaffold, promoting the binding between p-PLK1 and AURKA, thereby promoting MYC phosphorylation and enhancing the drug resistance [71]. Additional research is necessary to explore the impact of MYC-destabilizing lncRNAs.

## LncRNAs affecting the transcriptional activity of MYC

The primary function of MYC involves its role as a transcription factor with its recruitment to gene promoters and other regulatory regions required to activate or repress transcription [72]. In this capacity, the stabilized complex of LINC01600/EIF2S2/MYC as described above, is recruited to occupy an enhancer region in the *FHIT* gene, serving to repress gene activation and lifting its inhibition of Wnt/β-catenin pathway to promote CRC tumorigenesis [65]. In a similar fashion, the direct binding of the lncRNA MILNR1 to the MYC protein alters its transactivation of *NUP88*. Interestingly, MILNR1 is normally downregulated in CRC cells with its gene occurring close to *NUP88*. Among neighboring genes, MILNR1 appears to selectively inhibit *NUP88* transcription by preventing MYC recruitment to the *NUP88* promoter [73]. These findings indicate that lncRNAs may play a significant role in modulating MYC activity by influencing its participation in the transcription complex.

## MYC-related lncRNAs with different functions in different cancer types

While summarizing these lncRNAs in CRC, we broadened our scope to other cancer models and the known lncRNAs involved, surprisingly, we observed that some MYC-related lncRNAs may have different functions in different tumor types [74]. For instance, in the human B cell line P496-3, a MYC-regulated lncRNA LNROP (long noncoding regulator of POU2F2), was identified and shown to promote proliferation by acting as a cis-acting regulator of POU2F2 [75]. PHAROH (Pluripotency and Hepatocyte Associated RNA Overexpressed in HCC), can modulate MYC translation by interacting with and sequestering the translation repressor nucleolysin TIAR in hepatocellular carcinoma [76]. AFAP1-AS1, a lncRNA with high expression in lung cancer patients, is reported to promote cell invasion and migration by interacting with SNIP1 and inhibiting MYC ubiquitination [77]. In gastric cancer, LINC00942 binds with MSI2 and prevents its ubiquitination, whereas the overexpression of MSI2 can lead to the stabilization of *MYC* mRNA, which in turn promotes chemoresistance [78].

## Therapeutic prospects for targeting lncRNA/MYC axes

MYC represents an important and well-established predictor and treatment target for CRC. Indeed, many studies have focused on MYC targeting to replace or augment regular treatments involving surgery, chemotherapy, radiotherapy, and immunotherapy [79–82]. Nevertheless, MYC has proven an elusive target with previous generations of inhibitors not proving suitable for clinical use. Currently, three drugs (Omomyc/PC-002/WBC100) have progressed to clinical research, while several others are in the preclinical development phase. The drug formulations encompass a variety of types, such as small molecule inhibitors, peptides, Protac, and molecular gels. Omomyc, a 91-amino acid-engineered peptide bearing a mutated leucine zipper domain of MYC, has been shown to effectively bind to endogenous MYC, inhibiting the interaction between the MYC-MAX complex and DNA-Ebox. Additionally, Omomyc can bind to the MAX protein, disrupting the MYC-MAX interaction and subsequently facilitating the degradation of endogenous MYC protein. This dual mechanism ultimately leads to the inhibition of MYC downstream gene transcription, highlighting Omomyc’s potential therapeutic utility [83]. OMO-103, a cell-penetrating peptide originating from Omomyc, was the subject of a phase I clinical trial that commenced in April 2021 to assess its safety, pharmacokinetics, and efficacy in treating advanced solid tumors (NCT04808362). Findings from the study indicated an extended presence of OMO-103 in human blood, implying a potential for prolonged retention within human tumors [84]. PC-002, a first-class deubiquitinase (DUB) inhibitor that induces MYC degradation, also referred to as Sepantronium bromide or YM155, potently blocks BIRC5 expression to specifically induce apoptosis in MYC-dependent tumor cells by targeting the degradation of MYC protein [85]. Currently, it is undergoing a phase 2 study for high-grade B cell lymphoma (NCT05263583). The novel oral active molecule WBC100, currently undergoing phase I clinical study (CTR20211600), specifically targets the nuclear localization signal 1 (NLS1)-Basic-nuclear localization signal 2 (NLS2) region of MYC. This precise targeting mechanism leads to the degradation of MYC protein through the ubiquitin E3-ligase STUB1-mediated 26S proteasome pathway, ultimately inducing apoptosis in cancer cells and highlighting its significant role in cancer treatment [86]. Recent progress appears more promising with a compound named 4-(3,5-dimethoxy-4-(((4-methoxyphenethyl) amino) methyl) phenoxy)-N-phenylaniline can effectively inhibit MYC/MAX dimerization and DNA binding, thus inducing apoptosis and inhibiting cell cycle of CRC cells [87]. MYC inhibitor 10058-F4 can inhibit the growth of CRC cells by down-regulating MYC, CIP2A, and their downstream anti-apoptotic proteins [88]. The combination of BET-Bromodomain inhibitor JQ1 and small molecule inhibitor ABT-263 inhibited MYC protein levels and MYC-driven miR-1271-5p expression, and induced CRC cell apoptosis through PMAIP1/MCL1 pathway [89]. In addition, the anticancer activity of RV59 against human CRC cancer cell lines has been demonstrated, with the MYC/CXCL8/TIMP1 signaling pathway being its functional target [90]. The above-mentioned drugs targeting MYC were summarized in Table 4. Thus, inhibitors targeting MYC are expected to become new therapeutic drugs for CRC, but this may require combinations with other agents to maximize their effectiveness [91]. In this regard, lncRNAs may fill this niche.

**Table 4:** Drugs targeting MYC.

Drugs targeting MYC	Function	Mechanism	Ref.
Omomyc/OMO-103	Inducing apoptosis	Inhibiting interaction between MYC-MAX complex and DNA-Ebox	[83, 84]
PC-002	Inducing apoptosis	Targeting the degradation of MYC protein.	[85]
WBC100	Inducing apoptosis	Targeting the nuclear localization signal 1 (NLS1)-Basic-nuclear localization signal 2 (NLS2) region of MYC.	[86]
4-(3,5-dimethoxy-4-(((4-methoxyphenethyl) amino) methyl) phenoxy)-N-phenylaniline	Apoptosis and inhibiting cell cycle	Inhibiting MYC/MAX dimerization and DNA binding	[87]
10058-F4	Inducing apoptosis	Down-regulating MYC, CIP2A and their downstream anti-apoptotic proteins	[88]
JQ1 and ABT-263	Inducing apoptosis	Inhibiting MYC protein level and inducing CRC cell apoptosis through PMAIP1/MCL1 pathway	[89]
RV59	Inducing apoptosis	MYC/CXCL8/TIMP1 signaling pathway	[90]

Indeed, the potential of lncRNAs as biomarkers for CRC diagnosis and prognosis is recognized but this needs further exploration [92]. As disclosed above, specific lncRNAs contribute to CRC tumorigenesis through mutual reciprocal interactions with MYC, opening new possibilities to counteract MYC and related pathways via lncRNA-targeted drugs. Notably, preclinical evidence supports this idea, for instance, altering lncRNA expression changes the sensitivity of CRC cells to 5-FU through the Wnt/β-catenin pathway. For example, lncRNA NEAT1 regulates cancer stemness through chromatin remodeling, thereby enhancing 5-FU resistance [46]. However, the feasibility of these therapies in vivo requires consideration of the pharmacology and tissue-specificity of these agents. Delivery methods such as liposomes, one of the most successful nanocarriers, have shown excellent performance in delivering targeted oncological treatment, improving the safety profile and therapeutic efficacy of encapsulated drugs [93]. For instance, an in vivo approach targeting lncRNA FLANC by administration of 1,2-dioleoyl-sn-glycero-3-phosphatidylcholine nanoparticles loaded with a specific small interfering RNA, induced a significant decrease in CRC metastases [94]. Unfortunately, there are currently no clinical cohort studies targeting lncRNAs, but further positive results in this preclinical space are expected to advance these ideas into the clinic. Targeting lncRNAs and MYC simultaneously can maintain the therapeutic effect while reducing the dosage of drugs, thereby attenuating side effects and avoiding drug resistance. Certainly, given the functional specificity of lncRNAs in different tumors, simultaneous targeting of specific lncRNAs with MYC in colorectal cancer is expected to improve the specificity of therapeutic effects.

A corollary to these ideas is which lncRNA would be an ideal target in CRC to antagonize MYC function? There are numerous options as we described above, with many MYC-regulated lncRNAs promoting key tumorigenic pathways while others are embedded in co-regulatory loops with MYC. Here, negative feedback loops could be exploited to inhibit MYC function, for example, enhancing the expression of lncRNA-MIF can degrade MYC by promoting the expression of the MYC-targeting E3-ligase FBXW7. Alternatively, the positive feedback loop established between lncRNA-MEF and MYC could conceivably be antagonized with dual approaches targeting lncRNA-MEF and MYC. Also, a MYC-regulated ovarian adenocarcinoma-amplified lncRNA (OVAAL), is reported to promote cell proliferation via a feedback control over MYC stability, thus, targeting OVAAL and MYC simultaneously is expected to produce a stronger tumor suppression effect [95]. Other ideas to consider, involve the modulation of ceRNA functions (miRNA sponging) between lncRNAs and *MYC* mRNA. For example, the lncRNA SNHG3 sponges miR-182-5p, releasing MYC from the regulatory control of miR-182-5p [96]. Given that miRNAs are diversely involved in the regulation of MYC at transcriptional and post-transcriptional levels [97, 98], it is likely that many other lncRNAs are also involved in this mode of regulation. Ultimately, the answer to this question will require further research but current knowledge already provides a list of targets for ready investigation.

## Conclusions and perspectives

**Fig. 3:**
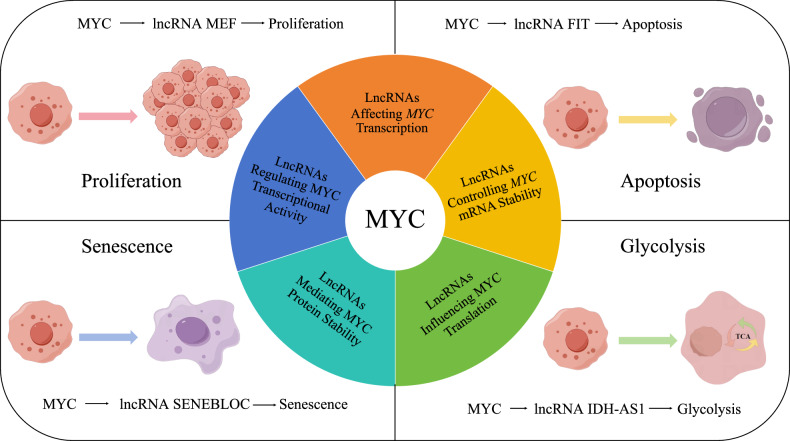
The interactions of lncRNAs and MYC in CRC. There are various reciprocal interactions between lncRNAs and MYC, and the following are several representative lncRNAs that have a regulatory relationship with MYC. MYC plays a crucial role in determining cell fate by regulating the expression of lncRNAs. For example, MYC can regulate the expression of lncRNA-MEF, which subsequently affects cell proliferation. Additionally, MYC can modulate apoptosis through its regulation of FIT. Moreover, IDH-AS1, a lncRNA responsive to MYC, has been found to bind to metabolic enzymes and impact glycolysis. SENEBLOC binding facilitates the association between p53 and MDM2, thereby regulating *CDKN1A* expression and ultimately influencing cell senescence. However, it is worth noting that certain lncRNAs can also reciprocally affect the function of MYC in various ways. LncRNAs can bind to transcription-associated proteins and influence MYC transcription. Besides, lncRNAs can exert their influence on translational regulation of MYC. Furthermore, lncRNAs are capable of affecting the stability of *MYC* mRNA and MYC protein. In addition to influencing expression level of MYC, lncRNAs have been shown to bind with MYC so as to influence its function of regulating gene transcription activities.

CRC represents a global public health burden, accounting for approximately 10% of all cancers diagnosed in 2022 and the second most frequent cause of cancer-related deaths. Here we described the latest discoveries, primarily in CRC, showing how lncRNAs impinge upon the MYC regulatory network, and disclosing the details of the multimodal regulation that occurs (summarized in Fig. 3). In this manner, we aimed to provide researchers with a better understanding and updated version of many key developmental mechanisms in CRC. Nevertheless, while our review focused on lncRNAs, we must acknowledge that other forms of ncRNAs such as circular RNAs (circRNAs) are also important regulators. For example, A newly discovered circRNA, circDNA2v, can bind to and prolong the half-life of IGF2BP3, thereby maintaining *MYC* mRNA levels [99]. CircRNAs have been a hot research topic in recent years, however, the functional intersections between circRNAs and MYC in CRC remain a work in progress. Nonetheless, circRNAs exhibit potential advantages for clinical applications, particularly their increased stability compared to lncRNAs which may help their utility as biomarkers.

The proto-oncogene *MYC* is reported to initiate tumor progression in most cancer types. Thus, accurate targeting of MYC is expected to provide more effective therapeutic options for many cancers including CRC. However, the lack of clinically suited inhibitors has limited this prospect, albeit recent research in the area brings this possibility closer to reality. A possible resolution, as proposed here, involves targeting interactions between MYC and lncRNAs. In this review, we set out to expose the various MYC-lncRNA networks that exist in CRC that provide the means to potentially target MYC through manipulating lncRNA expression. This approach also brings its own challenges, but delivery methods involving nanomedicines offer a promising means to realize this promise. We anticipate further advancements in this field, particularly the development of innovative approaches for CRC diagnosis and treatment that may benefit specific patients who could derive advantages from lncRNA-MYC regulation in CRC, thereby enhancing their quality of life and reducing medical expenses.
